# Brain Cortical and Hippocampal Dopamine: A New Mechanistic Approach for* Eurycoma longifolia* Well-Known Aphrodisiac Activity and Its Chemical Characterization

**DOI:** 10.1155/2019/7543460

**Published:** 2019-06-02

**Authors:** Shahira M. Ezzat, Marwa I. Ezzat, Mona M. Okba, Salah M. Hassan, Amgad I. Alkorashy, Mennatallah M. Karar, Sherif H. Ahmed, Shanaz O. Mohamed

**Affiliations:** ^1^Pharmacognosy Department, Faculty of Pharmacy, Cairo University, Kasr El-Ainy Street, Cairo 11562, Egypt; ^2^Pharmacognosy Department, Faculty of Pharmacy, October University for Modern Science and Arts (MSA), 6th of October 12566, Egypt; ^3^Department of Biochemistry, Faculty of Science, Ain Shams University, Cairo, Egypt; ^4^Department of Biochemistry, Faculty of Pharmacy, Al-Azhar University, Cairo, Egypt; ^5^Zewail City of Science and Technology, Cairo, Egypt; ^6^Department of Biochemistry, Faculty of Agriculture, Cairo University, Cairo, Egypt; ^7^School of Pharmaceutical Sciences, Universiti Sains Malaysia, Malaysia

## Abstract

*Eurycoma longifolia *Jack (Fam.: Simaroubaceae), known as Tongkat Ali (TA), has been known as a symbol of virility and sexual power for men. Metabolic profiling of the aqueous extract of* E. longifolia* (AEEL) using UPLC-MS/MS in both positive and negative modes allowed the identification of seventeen metabolites. The identified compounds were classified into four groups: quassinoids, alkaloids, triterpenes, and biphenylneolignans. AEEL is considered safe with oral LD_50_ cut-off >5000 mg/kg. Oral administration of 50, 100, 200, 400, or 800 mg/kg of AEEL for 10 consecutive days to Sprague-Dawley male rats caused significant reductions in mounting, intromission, and ejaculation latencies and increased penile erection index. AEEL increased total body weight and relative weights of seminal vesicles and prostate. Total and free serum testosterone and brain cortical and hippocampal dopamine content was significantly elevated in treated groups with no significant effects on serotonin or noradrenaline content.

## 1. Introduction

The World Health Organization defines infertility as the inability of a couple to bring a pregnancy to term after one year or more of regular, unprotected sexual intercourse or achieve conception [1]. Infertility is a major clinical concern, affecting 15% of all reproductive‐aged couples, and male factors are responsible for 25% of these cases [2]. The decline of male fertility has been highlighted as a serious public health issue in this century and associated with advancing age, incorrect lifestyles, and several environmental toxicants [3]; investigating alternative therapies to manage male infertility may prove cost‐effective and may provide the patient with a holistic approach to medicine.

*Eurycoma longifolia* Jack or “Tongkat Ali” is a herb which has been claimed to possess various medicinal properties. Traditionally, people believe that this herb can be used as remedies for sexual dysfunction, constipation, cancer, leukemia, exercise recovery, loss of libido, aging, stress, high blood pressure, malaria, osteoporosis, diabetes, fever, and glandular swelling [4].* E. longifolia* has been taken for its aphrodisiac properties for males [5]. In Malaysia, it was taken to improve strength and power during sexual activities [6].* E. longifolia* extracts lead to an increase in sexual arousal and motivation and frequency of sexual activity in both rats and mice [7].* E. longifolia* is famously known for its aphrodisiac effect, which is due to its ability to stimulate the production or action of androgen hormones, especially testosterone. Clinical and experimental studies indicate additional pharmacological activities of* E. longifolia. *The plant was shown to enhance immunity [8], improve quality of life and mood [9], protect against osteoporosis [10], and prevent obesity [11].

Phytochemical studies on this herb revealed that this herb possesses quassinoids which give the bitter taste [4]. These quassinoids include eurycomanone, eurycomalactone, laurycolactone, eurycolactone D, eurycolactone F, and eurycolactone E [6]. Pharmacological activity of this plant is attributed to these various quassinoids and also tirucallane-type triterpenes, squalene derivatives, biphenylneolignans, canthine-6-1, and beta carboline alkaloids [5].

However, studies investigating the* in vivo* effects of* E. longifolia* extract on male reproductive functions, especially its effects on spermatozoa, are limited to sperm concentration and motility or to the serum testosterone concentration. Therefore, this study aimed at investigating the effect of aqueous extract of* E. longifolia* (AEEL) in a broader manner on general well‐being, the brain cortical and hippocampal content of dopamine, serotonin, and noradrenaline. In addition, the parameters of sexual behavior (mount latency (ML), ejaculation latency (EL), intromission latency (IL), postejaculatory interval (PEI), and penile erection index), FSH, LH, free and total testosterone, and relative sex organ (testes, prostate, and seminal vesicles) to body weights were investigated. Furthermore a metabolic profiling of the aqueous extract was performed using UPLC-MS/MS.

## 2. Materials and Methods

### 2.1. Chemicals and Kits

AccuBind™ total testosterone and free testosterone ELISA kits were purchased from Monobind Inc., Lake Forest, CA, USA (CAT. #: 3725-300 and 5325-300, respectively). FSH, LH, and ELISA kits were acquired from BioCheck, Inc., CA, USA (CAT. #: BC-1029 and BC-1031, respectively). Dopamine, serotonin, and noradrenaline ELISA kits were obtained from Glory Science Co., Ltd, TX, USA (CAT. #: 90356, A1082, and 30587, respectively). All chemicals were of the highest commercial grade. The aqueous extract of* E. longifolia *was supplied by Technology Park Malaysia (TPM) Corporation Sdn. Bhd., Kuala Lumpur, Malaysia. Acetonitrile and Methanol were both of HPLC grade, purchased from Sigma Aldrich Chemie GmbH, Steinheim, Germany.

### 2.2. Plant Material

*Eurycoma longifolia *Jack roots were obtained from HCA products Sdn Bhd. Spring 2015. The plant was kindly identified in the Forest Research Institute, Malaysia. A voucher specimen (5-09-2015) was kept in the herbarium of Pharmacognosy Department, Faculty of Pharmacy, Cairo University, Cairo, Egypt.

### 2.3. Extraction

The dried powdered roots (1.5 kg) of* E. longifolia *were boiled with 10-liter distilled water for 15 min. and then kept for 1 h.; after that, the aqueous extract was filtered and lyophilized to obtain 800 g of pale brown powder of the aqueous extract of* E. longifolia *(AEEL).

### 2.4. UPLC-MS/MS Analysis of AEEL

Chromatographic separations were performed on an Agilent 6420 triple quad UPLC system (Agilent, California, USA) equipped with Acquity BEH shield reversed phase 18 column (1500 × 2.1 mm, particle size 1.7 *μ*m; Waters Milford, USA). The mobile phase was a binary solvent system consisting of solvent A (acetonitrile) and solvent B (water with 0.1% formic acid). The following were considered: the UPLC gradient at a flow rate of 0.3 ml/min: 0–5 min, isocratic 10 % B; 5–15 min, linear from 10 to 70 % B; 15–32 min, linear from 70 to 90 % B; 32–40 min, isocratic 90% B, 40–50 min, linear from 90 to 95 % B, 50–56 min, linear from 95 to 50 % B, 56–60 min, linear from 50 to 10 % B, isocratic 10% B, 61–70 min. The injection volume was 3.1 *μ*l. Eluted compounds were detected from m/z 100 to 1000 using a MS QQQ mass spectrometer equipped with an electrospray ion source in negative ion mode. Metabolites were characterized by their mass spectra, relative retention times, and comparison to literature.

### 2.5. Animals

Sprague–Dawley rats of both sexes, weighing from 200g to 250g, were purchased from the Animal Facility of Misr University for Science and Technology (MUST), 6^th^ of October City, Giza, Egypt. They were housed, females and males, separately in the animal facility at 50 ± 10% RH (Relative Humidity), 22 ± 3°C, and 12 h dark/light cycle. They were provided with pellet diet and water* ad libitum*. The animals were acclimatized to the housing environment for 7 days before dosing. The study was conducted in accordance with internationally accepted principles for laboratory animal use and care and was approved by Ethics Committee, Faculty of Pharmacy, Cairo University, Cairo, Egypt (MP 2161).

### 2.6. Determination of *LD*_50_

Determination of LD_50_ of AEEL was done according to OECD Guideline #423 (OECD; [12]) on Sprague-Dawley rats. Based on a previous pilot study in our laboratories, AEEL was administered orally to 3 animals using gastric feeding gavage at a dose of 2000 mg/kg (10 mL/kg dosing volume). The tested animals were observed for mortality after 24 h of administration. The test was repeated at the same dose using 3 extra animals. Thereafter, the animals were monitored daily for behavioral changes and weekly for changes in body weight. Necropsy was done on day 14 after administration of the single dose of the extract.

### 2.7. Evaluation of Aphrodisiac Activity

Male rats (48) were divided into 6 groups of 8 rats each. The control group received 3 mL/kg of water, whilst the other 5 groups received 50, 100, 200, 400, and 800 mg/kg of AEEL suspended in water as a single oral daily dose for 10 days. On day 11, male and female rats were mated and sexual behavior parameters along with total body weights were evaluated.

After 24 hours, blood samples were obtained from all male rats by retroorbital plexus puncture method for hormonal assessment under light anesthesia. Then, animals were killed by deep anesthesia with sodium pentobarbital and assessed for relative sex organ (testes, prostate, and seminal vesicles) to body weights and brain neurotransmitters content.

### 2.8. Sexual Behavior Test

The sexual behavior of males was observed by well-trained technicians, without knowing the study protocol, in an air conditioned, sound-attenuated room lit with a faint red light, amid the first period of the dark cycle of day 10. Single male rats were transferred into rectangular glass monitoring cages (40×50×40 cm) and allowed to get accommodated to the testing chamber for 15 min. Then, sexually receptive female rats were presented in the cages (1 female per cage) and the mating test began. The undermentioned parameters of sexual behavior were assessed as beforehand explained [13, 14]. Mount latency (ML) is defined as time (in seconds) from the introduction of the female to the first mount; ejaculation latency (EL) is defined as time (in seconds) from the first intromission to ejaculation; intromission latency (IL) is defined as time (in seconds) from introduction of the female to the first intromission (vaginal penetration); postejaculatory interval (PEI) is defined as time (in seconds) from ejaculation to the first intromission of the second copulatory series, and penile erection index = % rats exhibiting erection × mean number of erections.

### 2.9. Determination of FSH, LH, and Free and Total Testosterone

Investigating serum hormonal levels was performed by measuring FSH, LH, and total and free testosterone. Hormonal levels were quantified in the collected rat sera using the provided rat ELISA kits, according to the product instructions.

The assays of FSH and LH are based on sandwich ELISA technique using specific monoclonal antibody coated on a 96-well plate. A dose-response curve could be generated by using several different serum references of known antigen concentration. The antigen concentration of an unknown could be ascertained from this curve [15, 16].

### 2.10. Assessment of Dopamine, Serotonin, and Noradrenaline

Brains were rapidly removed and placed in ice-cold buffer, and brain regions were dissected and frozen on dry ice using procedures previously described [17, 18]. Brain neurotransmitters (dopamine, serotonin, and noradrenaline) content was quantified in the collected rat brain cortical and hippocampal tissues using the provided ELISA kits, according to the manufacturer instructions, based on the sandwich technique.

### 2.11. Statistical Analysis

Data are presented as mean ± SD. Statistical analysis was done using one-way ANOVA followed by Dunnett's post-hoc test for comparison of each treatment group and control. The 0.05 level of probability was used as the criterion for significance. All statistical analyses were done using GraphPad InStat software version 3 (La Jolla, CA, USA). Graphs were sketched using GraphPad Prism software version 5 (ISI® software, CA, USA).

## 3. Results

### 3.1. UPLC-MS/MS

The chemical composition of AEEL was examined using UPLC-MS/MS. All the metabolites were characterized by the interpretation of their mass spectra and the data provided by databases and literature.

**Figure 1:**
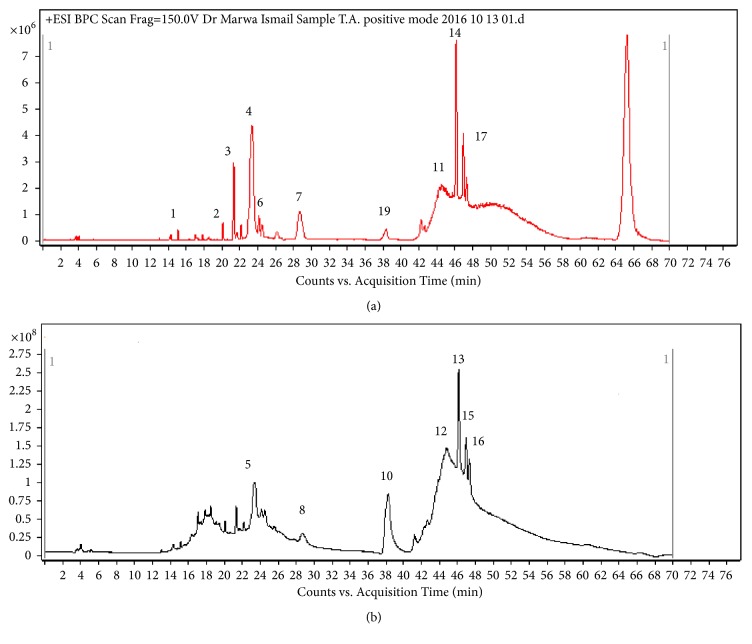
A representative UPLC-positive ionization MS trace (a) and negative ionization MS trace (b) of the aqueous extract of* E. longifolia* roots. Peak numbers follow those listed in Table 1 for metabolite identification using UPLC-MS/MS.

Seventeen compounds were identified using UPLC-MS/MS in both positive modes in which protonated and/or alkali adduct analyte molecules were generally observed in the mass spectra and negative modes where operation peaks corresponding to deprotonated analyte molecules are observed (Figures 1(a)-1(b); Table 1). Eight quassinoids were identified in the positive mode, and peak** 1** (Rt 15.01 min) showed a molecular ion peak [M+H]^+^ at* m/z* 351.100 which is corresponding to the molecular formula C_18_H_19_ClO_5_. The presence of a chlorine atom was confirmed by the appearance of an isotope peak [M+2]^+^ at* m/z* 352.000 in addition to a peak at 315.010 corresponding to [(M+H)-HCl]^+^. This compound was identified as Eurycolactone B [19]. Peak** 2** (Rt 20.05 min) showed a molecular ion [M+H]^+^ at* m/z* 365.0000 which could be correspondent to the molecular formula C_19_H_24_O_7_ that produced MS^2^ fragment at* m/z* 347.000 and 318.900 equivalent to [(M+H)-H_2_O]^+^ and [(M+H)-CH_2_O_2_]^+^, respectively. This fragmentation pattern is characteristic to the quassinoid 6*α*-hydroxyeurycomalactone [20].

Mass data of peak** 3** (Rt 21.92 min) had a molecular ion peak [M+H]^+^ at* m/z *409.000 (C_20_H_24_O_9_) and showed a daughter ion at* m/z* 391.000 corresponding to the loss of a water molecule [(M+H)-H_2_O]^+^ and another ion at 345.100 corresponding to [(M+H)-CO]^+^. This compound was identified as Eurycomanone [21]. Peak** 4** (Rt 23.63 min) showed a molecular ion at* m/z* 413.200 and a MS^2^* m/z *395.100 and 376.800 with the loss of 18 and 36 amu corresponding to [(M+H)-H_2_O] and [(M+H)-2H_2_O], respectively. This compound was regarded as 13*β*,18-dihydroeurycomanol (C_20_H_28_O_9_) [20]. This compound was also detected in the negative mode as [M-H]^−^ at* m/z* 411.100.

Eurycomanol-2-*O*-*β*-D-glucopyranoside was assigned to peak** 6** (Rt 24.21 min) based on the MS; molecular ion at* m/z* 573.100 for molecular formula C_26_H_36_O_4_ and MS^2^* m/z* 375.200 due to the cleavage of sugar moiety and the loss of 2 water molecules. The daughter ion at* m/z* 375 lost another water molecule to give ion at* m/z* 357.000. The consecutive cleavage of a –CH_2_O– residue followed by the neutral loss of CO from product ion at* m/z* 357.000 led to the appearance of the fragment at* m/z* 299.100 [21], and this compound was also detected in the negative mode with [M-H]^−^ at* m/z* 571.100. Another quassinoid appeared at peak** 7** (Rt 29.54 min) that showed a molecular ion peak [M+3H]^+^ at* m/z* 383.200 with a daughter ion peak at* m/z* 365.100 [(M+3H)-H_2_O] due to the loss of a water molecule. This peak was identified as 15*β*-Hydroxyklaineanone [20]. Peak** 9** (Rt 38.36 min) also showed a molecular ion peak [M+H]^+^ at* m/z* 413.100 corresponding to the molecular formula C_20_H_28_O_9_; it showed MS^2^ at* m/z* 395.200 [(M+H)-H_2_O] that resulted from a loss of a water molecule; this quassinoid was identified as 5*α*, 14*β*, 15*β*-Trihydroxyklaineanone [20]. Peak** 11** (Rt 45.76 min) showed a molecular ion peak at* m/z *455.102 (C_22_H_30_O_10_) and gave daughter ions at* m/z *at 454.100 and 436.000 corresponding to M^+^ and [M-H_2_O]^+^. This compound was identified as 6*α*-Acetoxy-14,15*β*-dihydroxyklaineanone [22].

Moreover, five quassinoids were detected in the negative mode as peaks** 5**,** 8**,** 10**,** 12**, and** 13**. Peak** 5** (Rt 23.90 min) showed a molecular ion peak [M-H]^−^ at* m/z* 443.300 consistent with the molecular formula C_20_H_28_O_11_. This peak gave daughter ions at m/z 427.100 and 413.000 due to the loss of -CH and -CH_2_O, respectively. It was identified as 13*β*,21-dihydroxyeurycomanol. Peak** 8** (Rt 29.98 min) was identified as 14,15*β*-Dihydroxyklaineanone as it showed a molecular ion peak [M-H]^−^ at* m/z* 395.100 and MS^2^ at* m/z* 377.000 due to the loss of H_2_O (18 amu), 359.100 with the loss of 2H_2_O (36 amu), and 349.300 due to the loss of –CH_2_O_2_ [20]. Peak** 10** (Rt 38.43 min) was with a molecular ion at* m/z* 315.100 corresponding to the molecular formula C_18_H_20_O_5_ and had fragmentation ions at* m/z* 271.200 and 256.001 due to the loss of CO_2_ [(M-H)-CO_2_] and [(M-H)-CO_2_-CH_3_]. This fragmentation pattern was in consistence with that of Laurycolactone B [23]. Peak** 12** (Rt 45.91 min) was identified as (*α*/*β*-epoxide) Ailanthone (C_20_H_24_O_8_) with a base peak* m/z* 391.100 corresponding to [M-H]^−^ and MS^2^ at* m/z* 373.100 [(M-H)-H2O] and 363.200 [(M-H)-CO]. Peak** 13** (Rt 46.18 min) was with molecular ion at* m/z* 573.122 corresponding to the molecular formula C_26_H_38_O_14_ and was identified as Iandonoside B; it showed MS^2^ at* m/z* 555.000 [(M-H)-H2O], 513.900 [(M-H)-C_2_H_3_O_2_] [20].

Three alkaloids of cathin-6-one type were also detected: one was detected in the positive ionization mode as peak** 14** and two were detected in the negative ionization mode as peaks** 15** and** 16**. Peak** 14** (Rt 46.52 min) showed a molecular ion peak [M+H]^+^ at* m/z* 251.200 corresponding to the molecular formula C_15_H_10_O_2_N_2_. It showed also daughter ions at* m/z* 236.100 and 233.000 due to the peaks [(M+H)-CH_3_] and [(M+H)-H_2_O], respectively. This compound was identified as 9-methoxycanthin-6-one [20]. Peak** 15** (Rt 46.69 min) was with a molecular ion peak [M-H]^−^ at* m/z *279.000 corresponding to the molecular formula C_16_H_12_O_3_N_2_. MS^2^ appeared at m/z 264.000 [(M-H)-CH_3_], 251.100 [(M-H)-CO], and 237.000 [(M-H)-C_2_H_2_O]. This compound could be identified as 5,9-dimethoxycanthin-6-one [24]. Peak** 16** (Rt 47.45 min) had a molecular ion peak [M-H]^−^ at 265.100 corresponding to C_15_H_10_O_3_N_2_. Its daughter ions MS^2^ were detected at* m/z* 250.200 [(M-H)-CH_3_], 247.300 [(M-H)-H_2_O], and 222.200 [(M-H)-CHNO]; thus this was identified as 11-hydroxy-10-methoxycanthin-6-one [24].

**Figure 2:**
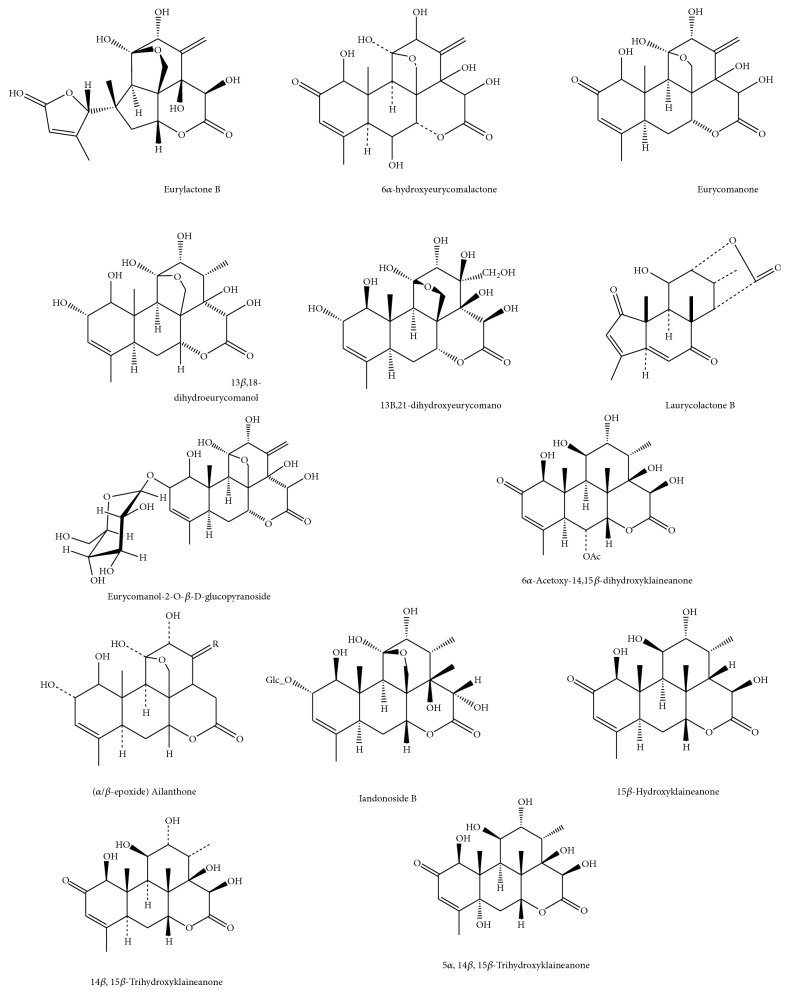
Structures of the identified quassinoids.

**Figure 3:**
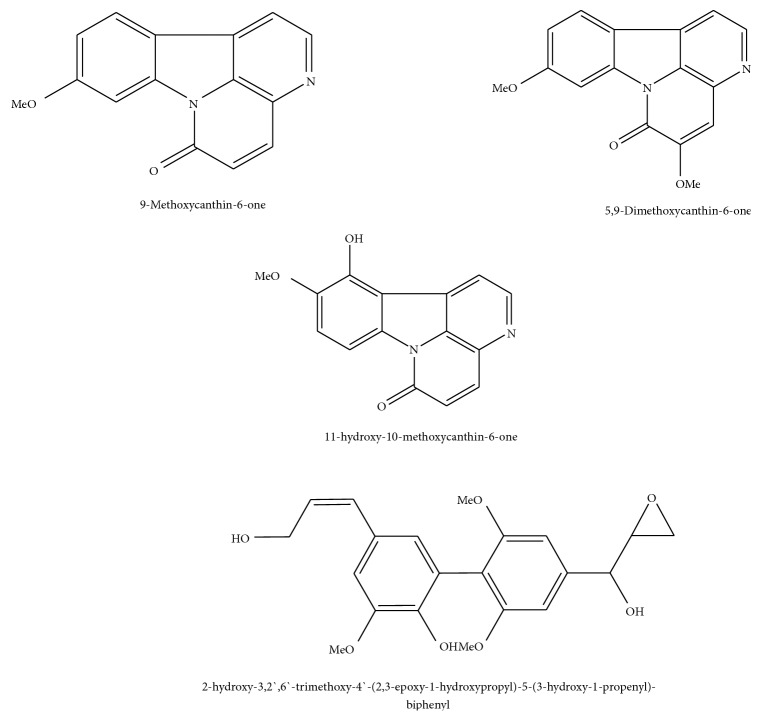
Structures of the identified alkaloids, triterpene, and biphenylneolignan.

Furthermore, a biphenylneolignan was observed in the positive ionization mode detected as Peak** 17** (Rt 47.91 min) that had a molecular ion peak [M+H]^+^ at 389.000 (C_21_H_24_O_7_) with MS^2^ at 371.110 [(M+H)-H_2_O], 357.001 [(M-H)-CH_4_O], and 343.100 [(M-H)-C_2_H_6_O] which was consistent with the biphenylneolignan;2-hydroxy-3,2′,6′-trimethoxy-4′-(2,3-epoxy-1-hydroxypropyl)-5-(3-hydroxy-1-propenyl)-biphenyl [22]. The identified compounds structures are presented in Figures 2 and 3.

### 3.2. Determination of *LD*_50_

A repeated single oral dose of AEEL (2000 mg/kg) did not show any mortality at 24 h after administration. Therefore,* E. longifolia* extract is considered unclassified according to the OECD with oral LD_50_ cut-off >5000 mg/kg.

### 3.3. Evaluation of Sexual Behavior

**Figure 4:**
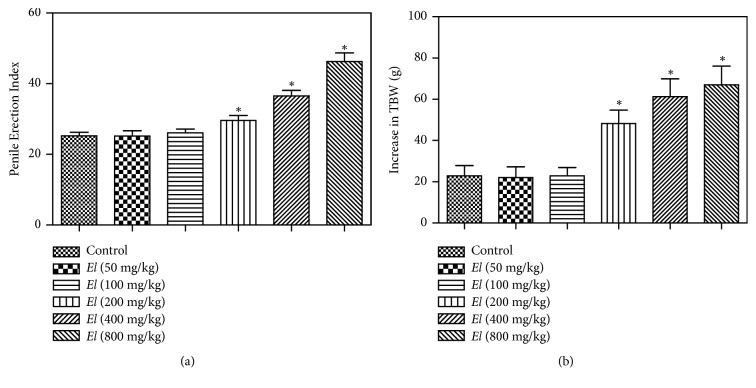
*Effect of E. longifolia root extract (a) on penile erection index of male rats and (b) on the increase of total body weight (TBW) of male rats. E. longifolia* was given as a single oral daily dose for 10 consecutive days. Values are mean ± SD. Statistical analysis was carried out by one-way* ANOVA* followed by* Dunnett post hoc* test. n=8. *∗*Significantly different from the control at p < 0.05. TBW = Total Body Weight;* EL = E. longifolia*.

Oral administration of AEEL (100, 200, 400, and 800 mg/kg) caused dose-related enhancement of male rat sexual behavior (Table 2). This was evidenced by significant reductions in ML, IL, and EL with doses 100, 200, 400, and 800 mg/kg as compared to the control group. Furthermore, the smallest dose (50 mg/kg) did not show significant changes in male rat behavior as compared to the control. However, no significant difference in PEI was recorded between control and treated animals. In addition, there was an improvement in male rats penile erection which was evidenced by a significant increase in penile erection index at the highest three doses (200, 400, and 800 mg/kg) compared to the respective control (Figure 4(a)).

### 3.4. Assessment of Body and Relative Organ-To-Body Weights

The oral administration of AEEL resulted in significant (2.1, 2.6, and 2.9-fold) increases in total body weight (TBW) of male rats at 200, 400, and 800 mg/kg, respectively (Figure 4(b)). Also, relative weights of prostate and seminal vesicles were increased significantly at doses of 400 and 800 mg/kg (Table 3). However, relative testicular weights were not significantly changed with AEEL administration.

### 3.5. Assessment of Serum FSH, LH, and Free and Total Testosterone

**Figure 5:**
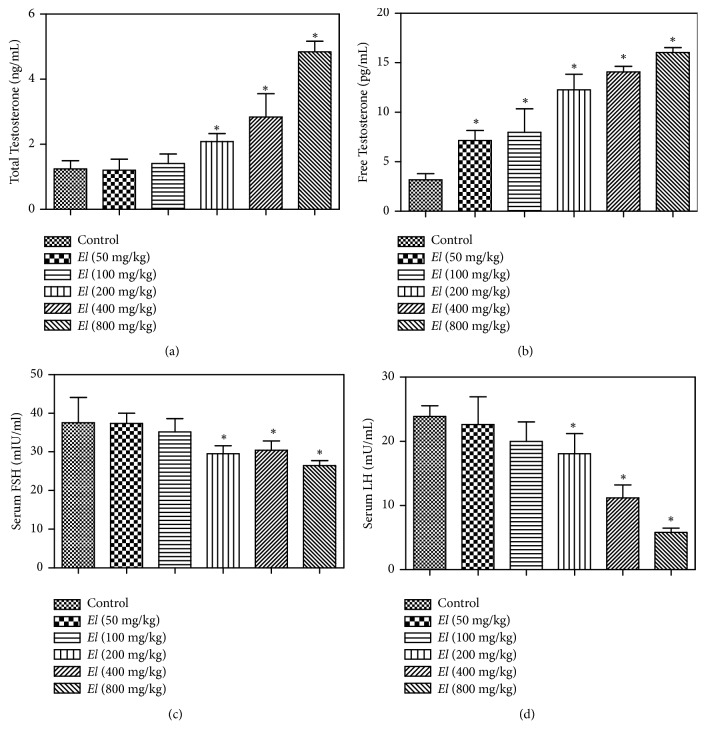
*Effect of E. longifolia root extract on serum levels of total testosterone (a), free testosterone (b), FSH (c), and LH (d) of male rats. E. longifolia* was given as a single oral daily dose for 10 consecutive days. Values are mean ± SD. Statistical analysis was carried out by one-way* ANOVA* followed by* Dunnett post hoc* test. n=8. *∗*Significantly different from the corresponding control at p < 0.05.* EL = E. longifolia*, FSH = Follicle Stimulating Hormone, and LH = Luteinizing Hormone.

Our data indicated that AEEL oral administration to male rats leads to significant elevation of total testosterone serum level by 67.1%, 127.8%, and 288.9% of control level at 200, 400, and 800 mg/kg, respectively (Figure 5(a)). Interestingly, serum level of free testosterone was significantly elevated compared to the corresponding control, starting from the lowest dose (2.2-fold at 50 mg/kg) and reaching 5-fold at the highest dose (800 mg/kg) (Figure 5(b)).

A reverse pattern was observed with FSH and LH levels. There were significant decreases in both FSH and LH levels at higher doses (200, 400, and 800 mg/kg), as compared to the corresponding control values. Serum levels of FSH were decreased by 21.4%, 18.9%, and 29.5% of the control level at 200, 400, and 800 mg/kg, respectively (Figure 5(c)). The decline in serum level of LH was more prominent than that of FSH. It was decreased by 24.3%, 53.2%, and 75.6% of its corresponding control values at the same doses, respectively (Figure 5(d)).

### 3.6. Evaluation of Brain Cortical and Hippocampal Dopamine, Serotonin, and Noradrenaline

**Figure 6:**
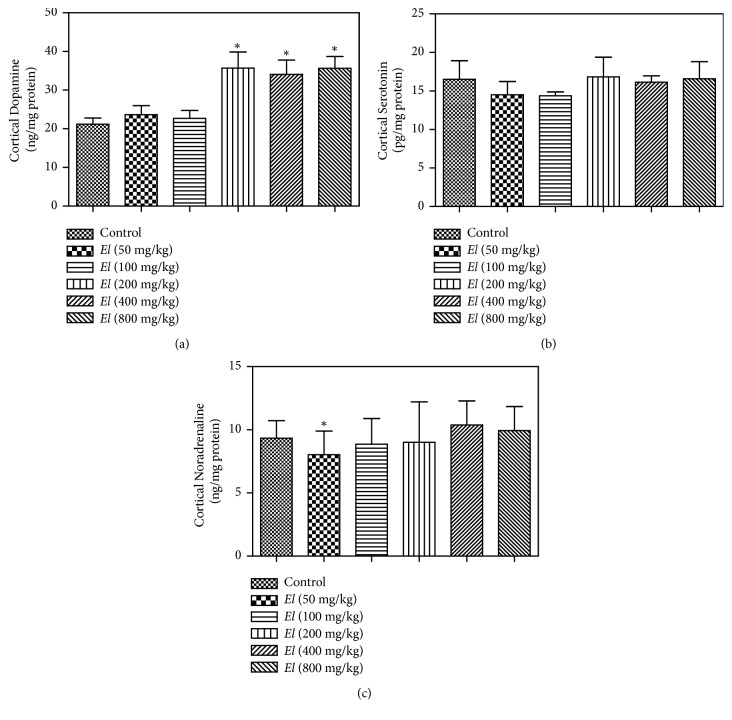
*Effect of E. longifolia root extract on cortical content of dopamine (a), serotonin (b), and noradrenaline (c) in male rats*.* E. longifolia *was given as a single oral daily dose for 10 consecutive days. Values are mean ± SD. Statistical analysis was carried out by one-way* ANOVA *followed by* Dunnett post hoc *test. n=8. **∗**Significantly different from the corresponding control at p < 0.05.* EL *=* E. longifolia.*

**Figure 7:**
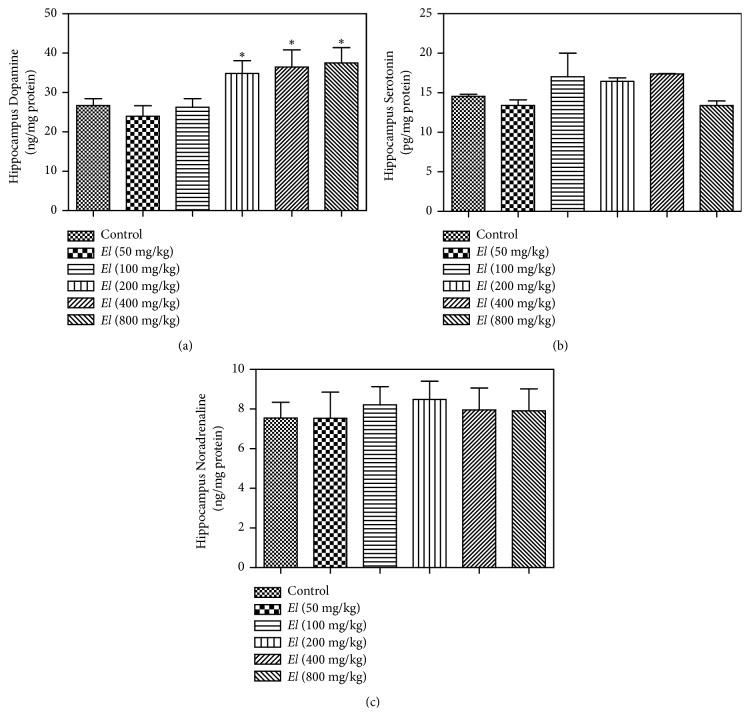
*Effect of E. longifolia roots on hippocampal content of dopamine (a), serotonin (b), and noradrenaline (c) in male rats*.* E. longifolia *was given as a single oral daily dose for 10 consecutive days. Values are mean ± SD. Statistical analysis was carried out by one-way* ANOVA *followed by* Dunnett post hoc *test. n=8. **∗**Significantly different from the corresponding control at p < 0.05.* EL *=* E. longifolia*.

Our evaluation indicated that AEEL oral administration to male rats resulted in significant elevation of cortical dopamine level at 200, 400, and 800 mg/kg (Figure 6(a)). However, no apparent effect was detected on cortical serotonin or noradrenaline levels (Figures 6(b) and 6(c)), respectively. The same pattern was recorded with hippocampal content of dopamine, serotonin, and noradrenaline. Hippocampal dopamine was significantly elevated at the higher doses (200, 400, and 800 mg/kg) (Figure 7(a)), while no significant change was observed with serotonin or noradrenaline content (Figures 7(b) and 7(c)), respectively.

## 4. Discussion

In Malaysia,* E. longifolia* has been reputed by Malays as a traditional remedy used as an adaptogen for energy and vitality and is well known for its aphrodisiac activities [25]. Although traditional use of* E. longifolia* as an aphrodisiac herb is well-recognized, there is a paucity of information on the possible underlying mechanisms. Therefore, the present study was conducted to substantiate the aphrodisiac activity of* E. longifolia.*

Initially, metabolic profiling of AEEL was performed using UPLC-MS/MS. A previous study was conducted which involved the LC-MS/MS analysis of the aqueous extracts of* E. longifolia *to discriminate between two samples cultivated in two different locations in Malaysia using the positive ionization mode [20]. Here we reported the identification of eighteen compounds using UPLC-MS/MS in both modes. The identified compounds could be classified into four groups: thirteen quassinoids, three alkaloids, a triterpene, and a biphenylneolignan.

LD_50_ of AEEL was determined in Sprague-Dawley rats according to OECD Guideline #423(OECD) (Supplementary File (available here)).* E. longifolia* was found to be safe and unclassified with oral LD_50_ cut-off >5000 mg/kg. This is consistent with the previous studies which indicated the same oral LD_50_ [4]. Administration of* E. longifolia* (50-800 mg/kg) to male rats resulted in obvious improvement of sexual behavior. This is evidenced by significant reductions in mounting, intromission, and ejaculation latencies and significant increase in penile erection index, at high dose levels. These findings gain support by several studies highlighting the aphrodisiac activities of* E. longifolia* which indicated an improvement in all parameters of sexual behavior towards receptive females [26–28]. In addition, the present study indicated that AEEL exhibits a significant anabolic effect, manifested by increased total body weight as well as relative seminal vesicles and prostate weights. This is in line with a previous study that reported that* E. longifolia* promoted the growth of both rat ventral prostate and seminal vesicles [5].

Assessment of the impact of AEEL on the pituitary-gonadal axis indicated significant elevation of the serum levels of total and free testosterone. This was confirmed by other studies which showed an increase in testosterone concentration after treatment of male rats with AEEL [29]. Rationally, a compensatory decline in FSH and LH levels was observed at the same dose range. This could be explained by the negative feedback inhibition mechanism controlling the internal endocrine environment. The elevated testosterone level explains the observed increase in sexual desire. This also provides an additional justification for the observed AEEL anabolic effect and the increase of sex organs weight.

Due to their key roles in behavioral functions including sexual behavior, the brain cortical and hippocampal contents of dopamine, serotonin, and noradrenaline were evaluated. Brain cortex and hippocampus are important structures in the sexual reward system [30, 31]. Dopamine plays a major role in most types of reward-motivated behavior including sexual reward [31], and serotonin is thought to be a contributor to feelings of happiness and well-being [32], while noradrenaline increases arousal and attentiveness and endorses vigilance [33]. Interestingly, AEEL administration was able to elevate dopamine but not serotonin or noradrenaline contents. Most types of reward, including sexual reward, involve an increase in brain dopamine. This is supported by the observed hypersexuality associated with dopamine-enhancing antiparkinsonian therapy [34]. Further, adverse effects of dopaminergic antagonist antipsychotics include decreased libido [35].

Collectively, the observed enhancement of free testosterone levels by* E. longifolia* was reflected on the possible negative feedback actions on LH and FSH levels as well as modulation of related brain neurotransmitters. Androgens modulate male sexual behavior and act at both the central and peripheral nervous system levels [36]. The testosterone-induced enhancement of dopamine release and its impact on the control of sexual behavior has been previously described. The stimuli from a receptive female lead to the release of dopamine in different brain areas. These include the nigrostriatal system, the mesolimbic system, and the medial preoptic area. The previous presence of testosterone is permissive for dopamine release and increases copulatory rate and efficiency and coordinates genital reflexes [37].

The positive effect of* E. longifolia* in the improvement of sexual behavior may be attributed to its active constituents such as quassinoids and in particular the major one, eurycomanone which was detected as peak** 3** in UPLC-MS/MS analysis of AEEL. Eurycomanone was reported to induce testosterone production [4] and was also reported to enhance testosterone steroidogenesis at the Leydig cells through its inhibitory effect on the final step of transformation of testosterone to estrogen through aromatase enzyme inhibition [38]. Moreover, high concentration of eurycomanone has inhibitory effect on phosphodiesterase [38].

## 5. Conclusion

The current data confirm the aphrodisiac and anabolic activities of* E. longifolia* roots aqueous extract in male Sprague-Dawley rats. This can be attributed, at least partly, to elevation of blood testosterone level as well as enhancement of brain cortical and hippocampal dopamine content.

## Figures and Tables

**Table 1:** Peak assignments of *E. longifolia* aqueous extract metabolites using UPLC-MS/MS in positive and negative ionization modes.

Peak	t_R_ (min)	[M-H]^−^	[M+H]^−^	Molecular formula	Identification	MS^2^
1	15.01		351.100	C_18_H_19_ClO_5_	Eurycolactone B	352.000, 315.010, 290.800

2	20.05		365.000	C_19_H_24_O_7_	6*α*-Hydroxyeurycomalactone	347.000, 318.900, 202.100

3	21.92		409.000	C_20_H_24_O_9_	Eurycomanone	391.000, 373.200, 345.100, 225.000

4	23.63	411.100	413.200	C_20_H_28_O_9_	13*β*,18-dihydroeurycomanol	395.100, 376.800, 319.000, 291.200

5	23.90	443.300		C_20_H_28_O_11_	13*β*,21-dihydroxyeurycomanol	427.100, 413.000, 379.300

6	24.21	571.100	573.100	C_26_H_36_O_4_	Eurycomanol-2-O-*β*-D-glucopyranoside	375.200, 357.000, 299.100

7	29.54		383.200	C_20_H_28_O_7_	15*β*-Hydroxyklaineanone	365.100, 326.800, 266.000, 202.100

8	29.98	395.100		C_20_H_28_O_8_	14,15*β*-Dihydroxyklaineanone	377.000, 359.100, 349.300

9	38.36		413.100	C_20_H_28_O_9_	5*α*, 14*β*, 15*β*-Trihydroxyklaineanone	395.200

10	38.43	315.100		C_18_H_20_O_5_	Laurycolactone B	271.200, 256.001

11	45.76		455.102	C_22_H_30_O_10_	6*α*-Acetoxy-14,15*β*-dihydroxyklaineanone	454.100, 436.000

12	45.91	391.100		C_20_H_24_O_8_	(*α*/*β*-epoxide) Ailanthone	373.100, 363.200

13	46.18	573.122		C_26_H_38_O_14_	Iandonoside B	555.000, 513.900

14	46.52		251.200	C_15_H_10_O_2_N_2_	9-Methoxycanthin-6-one	236.100, 233.000, 216.200

15	46. 69	279.000		C_16_H_12_O_3_N_2_	5,9-Dimethoxycanthin-6-one	264.000, 251.100, 237.000

16	47.45	265.100		C_15_H_10_O_3_N_2_	11-Hydroxy-10-methoxycanthin-6-one	250.200, 247.300, 222.200

17	47.91		389.000	C_21_H_24_O_7_	2-Hydroxy-3,2′,6′-trimethoxy-4′-(2,3-epoxy-1-hydroxypropyl)-5-(3-hydroxy-1-propenyl)-biphenyl	371.110, 357.001, 343.100, 295.000

**Table 2:** Effect of *E. longifolia* root extract on male rats sexual behavior.

Group	Dose (mg/kg)	ML (sec.)	IL (sec.)	EL (sec.)	PEI (sec.)
Control		373.6 ± 6.56	525.6 ± 6.65	1045.8 ± 15.3	304 ± 4.34

AEEL	50	355.8 ± 21.77	482.5^*∗*^ ± 21.15	975.8^*∗*^ ± 25.18	298.5 ± 14.77

	100	280.3^*∗*^ ± 4.98	410.5^*∗*^ ± 12.37	836.6^*∗*^ ± 17.21	301.3 ± 9.42

	200	192.3^*∗*^ ± 17.79	242.16^*∗*^ ± 16.49	542.5^*∗*^ ± 35.6	299.8 ± 8.84

	400	160.5^*∗*^ ± 7.68	169.5^*∗*^ ± 7.39	341.6^*∗*^ ± 7.96	301.5 ± 10.01

	800	143.39^*∗*^ ± 7.13	153.3^*∗*^ ± 5.35	296.6^*∗*^ ± 7.76	305.8 ± 7.11

*E. longifolia* was given as a single oral daily dose for 10 consecutive days. Values are mean ± SD. n=8.

*∗* Significantly different from the corresponding control at p < 0.05.

AEEL: aqueous extract of *E. longifolia; *EL = Ejaculation Latency, IL = Intromission Latency, ML = Mount Latency, and PEI = Post-Ejaculatory Interval.

**Table 3:** Effect of *E. longifolia* root extract on relative weights of testes, seminal vesicles, and prostate in male rats.

Group	Dose (mg/kg)	Testes/TBW	Seminal Vesicles/TBW	Prostate/TBW
Control		0.0067 ± 0.0004	0.0024 ± 0.0006	0.0015 ± 0.0004

AEEL	50	0.0074 ± 0.0009	0.0035 ± 0.0008	0.0016 ± 0.0004

	100	0.0079 ± 0.0014	0.0032 ± 0.0006	0.0016 ± 0.0002

	200	0.0079 ± 0.0011	0.0035 ± 0.0011	0.0020 ± 0.0003

	400	0.0075 ± 0.0012	0.0043^*∗*^ ± 0.0006	0.0024^*∗*^ ± 0.0004

	800	0.0079 ± 0.0005	0.0049^*∗*^ ± 0.0006	0.0026^*∗*^ ± 0.0003

*E. longifolia* was given as a single oral daily dose for 10 consecutive days. Values are mean ± SD. n=8.

**∗**Significantly different from the corresponding control at p < 0.05.

AEEL: aqueous extract of *E. longifolia;* TBW = Total Body Weight.

## Data Availability

The data used to support the findings of this study are included within the article.

## Supplementary Materials

Supplementary Materials OECD guidelines.

## References

[1] Rowe P. J., Comhaire F. H., Hargreave T. B., Mahmoud A. M. A., World Health Organization (2000). *WHO Manual for the Standardized Investigation, Diagnosis and Management of the Infertile Male*.

[2] Jarow J. P., Sharlip I. D., Belker A. M. (2002). Best practice policies for male infertility. *The Journal of Urology*.

[3] Ilacqua A., Izzo G., Emerenziani G. P., Baldari C., Aversa A. (2018). Lifestyle and fertility: the influence of stress and quality of life on male fertility. *Reproductive Biology and Endocrinology*.

[4] Rehman S., Choe K., Yoo H. (2016). Review on a traditional herbal medicine, eurycoma longifolia Jack (Tongkat Ali): its traditional uses, chemistry, evidence-based pharmacology and toxicology. *Molecules*.

[5] Ang H. H., Cheang H. S., Yusof A. P. M. (2000). Effects of Eurycoma longifolia Jack (Tongkat Ali) on the initiation of sexual performance of inexperienced castrated male rats. *Journal of Experimental Animal Science*.

[6] Ang H. H., Hitotsuyanagi Y., Fukaya H., Takeya K. (2002). Quassinoids from Eurycoma longifolia. *Phytochemistry*.

[7] Ang H. H., Lee K. L., Kiyoshi M. (2003). Eurycoma longifolia jack enhances sexual motivation in middle-aged male mice. *Journal of Basic and Clinical Physiology and Pharmacology*.

[8] He P., Dong Z., Wang Q., Zhan Q., Zhang M., Wu H. (2019). Structural characterization and immunomodulatory activity of a polysaccharide from *Eurycoma longifolia*. *Journal of Natural Products*.

[9] George A., Udani J., Abidin N. Z., Yusof A. (2018). Efficacy and safety of Eurycoma longifolia (Physta®) water extract plus multivitamins on quality of life, mood and stress: a randomized placebo-controlled and parallel study. *Food & Nutrition Research*.

[10] Jayusman P., Mohamed I., Alias E., Mohamed N., Shuid A. (2018). The effects of quassinoid-rich eurycoma longifolia extract on bone turnover and histomorphometry indices in the androgen-deficient osteoporosis rat model. *Nutrients*.

[11] Balan D., Chan K., Murugan D., AbuBakar S., Wong P. (2018). Antiadipogenic effects of a standardized quassinoids-enriched fraction and eurycomanone from *Eurycoma longifolia*. *Phytotherapy Research*.

[12] Tambi M. I., Imran M. K., Henkel R. R. (2012). Standardised water-soluble extract of Eurycoma longifolia, Tongkat ali, as testosterone booster for managing men with late-onset hypogonadism?. *Andrologia*.

[13] Malmnäs C.-O., Meyerson B. J. (1971). P-chlorophenylalanine and copulatory behaviour in the male rat. *Nature*.

[14] Bell C. (1995). Clinical guide to laboratory tests. 3rd edition. Norbert W. Tietz, ed. *Transfusion*.

[15] Smith S. W. (1993). Free testosterone. *AACC Endo*.

[16] Chan K., Choo C. (2002). The toxicity of some quassinoids from eurycoma longifolia. *Planta Medica*.

[17] Bowyer J. F., Harris A. J., Delongchamp R. R. (2004). Selective changes in gene expression in cortical regions sensitive to amphetamine during the neurodegenerative process. *NeuroToxicology*.

[18] Spijker S. (2011). Dissection of rodent brain regions. *Neuromethods*.

[19] Ang H. H., Hitotsuyanagi Y., Takeya K. (2000). Eurycolactones A-C, novel quassinoids from Eurycoma longifolia. *Tetrahedron Letters*.

[20] Chua L. S., Amin N. A., Neo J. C. (2011). LC-MS/MS-based metabolites of Eurycoma longifolia (Tongkat Ali) in Malaysia (Perak and Pahang). *Journal of Chromatography B*.

[21] Teh C.-H., Murugaiyah V., Chan K.-L. (2011). Developing a validated liquid chromatography-mass spectrometric method for the simultaneous analysis of five bioactive quassinoid markers for the standardization of manufactured batches of *Eurycoma longifolia* Jack extract as antimalarial medicaments. *Journal of Chromatography A*.

[22] Morita H., Kishi E., Takeya K., Itokawa H., Iitaka Y. (1993). Highly oxygenated quassinoids from Eurycoma longifolia. *Phytochemistry*.

[23] Mutschlechner B., Schwaiger S., Tran T. V. A., Stuppner H. (2018). Development of a selective HPLC-DAD/ELSD method for the qualitative and quantitative assessment of commercially available Eurycoma longifolia products and plant extracts. *Fitoterapia*.

[24] Mitsunaga K., Koike K., Tanaka T. (1994). Canthin-6-one alkaloids from *Eurycoma longifolia*. *Phytochemistry*.

[25] Bhat R., Karim A. A. (2010). Tongkat Ali (*Eurycoma longifolia* Jack): a review on its ethnobotany and pharmacological importance. *Fitoterapia*.

[26] Ang H. H., Lee K. L. (2002). Effect of *Eurycoma longifolia* Jack on orientation activities in middle-aged male rats. *Fundamental & Clinical Pharmacology*.

[27] Ang H. H., Ngai T. H., Tan T. H. (2003). Effects of Eurycoma longifolia Jack on sexual qualities in middle aged male rats. *Phytomedicine*.

[28] Ang H. H., Lee K. L., Kiyoshi M. (2004). Sexual arousal in sexually sluggish old male rats after oral administration of eurycoma longifolia jack. *Journal of Basic and Clinical Physiology and Pharmacology*.

[29] Solomon M. C., Erasmus N., Henkel R. R. (2014). *In vivo* effects of *Eurycoma longifolia* Jack (Tongkat Ali) extract on reproductive functions in the rat. *Andrologia*.

[30] Pfaus J. G., Scepkowski L. A. (2005). The biologic basis for libido. *Current Sexual Health Reports*.

[31] Schultz W. (2015). Neuronal reward and decision signals: from theories to data. *Physiological Reviews*.

[32] Young S. N. (2007). How to increase serotonin in the human brain without drugs. *Journal of Psychiatry & Neuroscience*.

[33] Berridge C. W., Schmeichel B. E., España R. A. (2012). Noradrenergic modulation of wakefulness/arousal. *Sleep Medicine Reviews*.

[34] Bronner G., Aharon-Peretz J., Hassin-Baer S. (2015). Sexuality in patients with Parkinson's disease, Alzheimer's disease, and other dementias. *Handbook of Clinical Neurology*.

[35] Just M. J. (2015). The influence of atypical antipsychotic drugs on sexual function. *Neuropsychiatric Disease and Treatment*.

[36] Isidori A. M., Buvat J., Corona G. (2014). A critical analysis of the role of testosterone in erectile function: from pathophysiology to treatment—a systematic review. *European Urology*.

[37] Hull E. M., Lorrain D. S., Du J. (1999). Hormone-neurotransmitter interactions in the control of sexual behavior. *Behavioural Brain Research*.

[38] Low B.-S., Choi S.-B., Abdul Wahab H., Kumar Das P., Chan K.-L. (2013). Eurycomanone, the major quassinoid in Eurycoma longifolia root extract increases spermatogenesis by inhibiting the activity of phosphodiesterase and aromatase in steroidogenesis. *Journal of Ethnopharmacology*.

