# Supplementary material for: Brain Cortical and Hippocampal Dopamine: A New Mechanistic Approach for Eurycoma longifolia Well-Known Aphrodisiac Activity and Its Chemical Characterization
Source: Evid Based Complement Alternat Med. 2019 Jun 2;2019:7543460. doi: 10.1155/2019/7543460 (PMC6582863; doi:10.1155/2019/7543460)
Supplement: Supplementary Materials — OECD guidelines. [file 7543460.f1.pdf]

Adopted:
17th December 2001

OECD GUIDELINE FOR TESTING OF CHEMICALS

Acute Oral Toxicity – Acute Toxic Class Method

INTRODUCTION

1. OECD Guidelines for the Testing of Chemicals are periodically reviewed in the light of scientific progress or changing assessment practices. The original Guideline 423 was adopted in March 1996 as the second alternative to the conventional acute toxicity test, described in Test Guideline 401. Based on the recommendations of several expert meetings, revision was considered timely because: i) international agreement has been reached on harmonised LD50 cut-off values for the classification of chemical substances, which differ from the cut-offs recommended in the 1996 version of the Guideline, and ii) testing in one sex (usually females) is now considered sufficient.

2. The acute toxic class method (1) set out in this Guideline is a stepwise procedure with the use of 3 animals of a single sex per step. Depending on the mortality and/or the moribund status of the animals, on average 2-4 steps may be necessary to allow judgement on the acute toxicity of the test substance. This procedure is reproducible, uses very few animals and is able to rank substances in a similar manner to the other acute toxicity testing methods (Test Guidelines 420 and 425). The acute toxic class method is based on biometric evaluations (2)(3)(4)(5) with fixed doses, adequately separated to enable a substance to be ranked for classification purposes and hazard assessment. The method as adopted in 1996 was extensively validated *in vivo* against LD50 data obtained from the literature, both nationally (6) and internationally (7).

3. Guidance on the selection of the most appropriate test method for a given purpose can be found in the Guidance Document on Acute Oral Toxicity Testing (8). This Guidance Document also contains additional information on the conduct and interpretation of Test Guideline 423.

4. Definitions used in the context of this Guideline are set out in Annex 1.

INITIAL CONSIDERATIONS

5. Test substances, at doses that are known to cause marked pain and distress due to corrosive or severely irritant actions, need not be administered. Moribund animals, or animals obviously in pain or showing signs of severe and enduring distress shall be humanely killed, and are considered in the interpretation of the test results in the same way as animals that died on test. Criteria for making the decision to kill moribund or severely suffering animals, and guidance on the recognition of predictable or impending death, are the subject of a separate Guidance Document (9).

6. The method uses pre-defined doses and the results allow a substance to be ranked and classified according to the Globally Harmonised System for the classification of chemicals which cause acute toxicity (10).

7. In principle, the method is not intended to allow the calculation of a precise LD_{50}, but does allow for the determination of defined exposure ranges where lethality is expected since death of a proportion of the animals is still the major endpoint of this test. The method allows for the determination of an LD50 value only when at least two doses result in mortality higher than 0% and lower than 100%. The use of a selection of pre-defined doses, regardless of test substance, with classification explicitly tied to number of animals observed in different states improves the opportunity for laboratory to laboratory reporting consistency and repeatability.

8. The testing laboratory should consider all available information on the test substance prior to conducting the study. Such information will include the identity and chemical structure of the substance; its physico-chemical properties; the result of any other *in vivo* or *in vitro* toxicity tests on the substance; toxicological data on the structurally related substances; and the anticipated use(s) of the substance. This information is necessary to satisfy all concerned that the test is relevant for the protection of human health and will help in the selection of the most appropriate starting dose.

PRINCIPLE OF THE TEST

9. It is the principle of the test that, based on a stepwise procedure with the use of a minimum number of animals per step, sufficient information is obtained on the acute toxicity of the test substance to enable its classification. The substance is administered orally to a group of experimental animals at one of the defined doses. The substance is tested using a stepwise procedure, each step using three animals of a single sex (normally females). Absence or presence of compound-related mortality of the animals dosed at one step will determine the next step, i.e.;

- no further testing is needed,
- dosing of three additional animals, with the same dose
- dosing of three additional animals at the next higher or the next lower dose level.

10. Details of the test procedure are described in Annex 2. The method will enable a judgement with respect to classifying the test substance to one of a series of toxicity classes defined by fixed LD50 cut-off values.

DESCRIPTION OF THE METHOD

Selection of animal species

11. The preferred rodent species is the rat, although other rodent species may be used. Normally females are used (9). This is because literature surveys of conventional LD50 tests show that, although there is little difference in sensitivity between the sexes, in those cases where differences are observed females are generally slightly more sensitive (11). However if knowledge of the toxicological or toxicokinetic properties of structurally related chemicals indicates that males are likely to be more sensitive, then this sex should be used. When the test is conducted in males adequate justification should be provided.

12. Healthy young adult animals of commonly used laboratory strains should be employed. Females should be nulliparous and non-pregnant. Each animal, at the commencement of its dosing, should be between 8 and 12 weeks old and its weight should fall in an interval within ± 20 % of the mean weight of any previously dosed animals.

Housing and feeding conditions

13. The temperature in the experimental animal room should be 22ºC ($\pm$ 3ºC). Although the relative humidity should be at least 30% and preferably not exceed 70% other than during room cleaning the aim should be 50-60%. Lighting should be artificial, the sequence being 12 hours light, 12 hours dark. For feeding, conventional laboratory diets may be used with an unlimited supply of drinking water. Animals may be group-caged by dose, but the number of animals per cage must not interfere with clear observations of each animal.

Preparation of animals

14. The animals are randomly selected, marked to permit individual identification, and kept in their cages for at least 5 days prior to dosing to allow for acclimatisation to the laboratory conditions.

Preparation of doses

15. In general test substances should be administered in a constant volume over the range of doses to be tested by varying the concentration of the dosing preparation. Where a liquid end product or mixture is to be tested however, the use of the undiluted test substance, ie at a constant concentration, may be more relevant to the subsequent risk assessment of that substance, and is a requirement of some regulatory authorities. In either case, the maximum dose volume for administration must not be exceeded. The maximum volume of liquid that can be administered at one time depends on the size of the test animal. In rodents, the volume should not normally exceed 1mL/100g of body weight: however in the case of aqueous solutions 2 mL/100g body weight can be considered. With respect to the formulation of the dosing preparation, the use of an aqueous solution/suspension/emulsion is recommended wherever possible, followed in order of preference by a solution/suspension/emulsion in oil (e.g. corn oil) and then possibly solution in other vehicles. For vehicles other than water the toxicological characteristics of the vehicle should be known. Doses must be prepared shortly prior to administration unless the stability of the preparation over the period during which it will be used is known and shown to be acceptable.

PROCEDURE

Administration of doses.

16. The test substance is administered in a single dose by gavage using a stomach tube or a suitable intubation canula. In the unusual circumstance that a single dose is not possible, the dose may be given in smaller fractions over a period not exceeding 24 hours.

17. Animals should be fasted prior to dosing (e.g. with the rat, food but not water should be withheld over-night, with the mouse, food but not water should be withheld for 3-4 hours). Following the period of fasting, the animals should be weighed and the test substance administered. After the substance has been administered, food may be withheld for a further 3-4 hours in rats or 1-2 hours in mice. Where a dose is administered in fractions over a period it may be necessary to provide the animals with food and water depending on the length of the period.

Number of animals and dose levels

18. Three animals are used for each step. The dose level to be used as the starting dose is selected from one of four fixed levels, 5, 50, 300 and 2000 mg/kg body weight. The starting dose level should be that which is most likely to produce mortality in some of the dosed animals. The flow charts of Annex 2 describe the procedure that should be followed for each of the starting doses.

19. When available information suggests that mortality is unlikely at the highest starting dose level (2000 mg/kg body weight), then a limit test should be conducted. When there is no information on a substance to be tested, for animal welfare reasons it is recommended to use the starting dose of 300 mg/kg body weight.

20 The time interval between treatment groups is determined by the onset, duration, and severity of toxic signs. Treatment of animals at the next dose, should be delayed until one is confident of survival of the previously dosed animals.

21. Exceptionally, and only when justified by specific regulatory needs, the use of additional upper dose level of 5000 mg/kg body weight may be considered (see Annex 3). For reasons of animal welfare concern, testing of animals in GHS Category 5 ranges (2000-5000mg/kg) is discouraged and should only be considered when there is a strong likelihood that results of such a test have a direct relevance for protecting human or animal health or the environment.

Limit test

22. The limit test is primarily used in situations where the experimenter has information indicating that the test material is likely to be nontoxic, i.e., having toxicity only above regulatory limit doses. Information about the toxicity of the test material can be gained from knowledge about similar tested compounds or similar tested mixtures or products, taking into consideration the identity and percentage of components known to be of toxicological significance. In those situations where there is little or no information about its toxicity, or in which the the test material is expected to be toxic, the main test should be performed.

23. A limit test at one dose level of 2000 mg/kg body weight may be carried out with six animals (three animals per step). Exceptionally a limit test at one dose level of 5000 mg/kg may be carried out with three animals (see Annex 3). If test substance-related mortality is produced, further testing at the next lower level may need to be carried out.

OBSERVATIONS

24. Animals are observed individually after dosing at least once during the first 30 minutes, periodically during the first 24 hours, with special attention given during the first 4 hours, and daily thereafter, for a total of 14 days, except where they need to be removed from the study and humanely killed for animal welfare reasons or are found dead. However, the duration of observation should not be fixed rigidly. It should be determined by the toxic reactions, time of onset and length of recovery period, and may thus be extended when considered necessary. The times at which signs of toxicity appear and disappear are important, especially if there is a tendency for toxic signs to be delayed (12). All observations are systematically recorded with individual records being maintained for each animal.

25. Additional observations will be necessary if the animals continue to display signs of toxicity. Observations should include changes in skin and fur, eyes and mucous membranes, and also respiratory, circulatory, autonomic and central nervous systems, and somatomotor activity and behaviour pattern. Attention should be directed to observations of tremors, convulsions, salivation, diarrhoea, lethargy, sleep

and coma. The principles and criteria summarised in the Humane Endpoints Guidance Document (9) should be taken into consideration. Animals found in a moribund condition and animals showing severe pain or enduring signs of severe distress should be humanely killed. When animals are killed for humane reasons or found dead, the time of death should be recorded as precisely as possible.

Body weight

26. Individual weights of animals should be determined shortly before the test substance is administered, and at least weekly thereafter. Weight changes should be calculated and recorded. At the end of the test surviving animals are weighed and humanely killed.

Pathology

27. All test animals (including those that die during the test or are removed from the study for animal welfare reasons) should be subjected to gross necropsy. All gross pathological changes should be recorded for each animal. Microscopic examination of organs showing evidence of gross pathology in animals surviving 24 or more hours may also be considered because it may yield useful information.

DATA AND REPORTING

Data

28. Individual animal data should be provided. Additionally, all data should be summarised in tabular form, showing for each test group the number of animals used, the number of animals displaying signs of toxicity, the number of animals found dead during the test or killed for humane reasons, time of death of individual animals, a description and the time course of toxic effects and reversibility, and necropsy findings.

Test report

29. The test report must include the following information, as appropriate:

Test substance:

- physical nature, purity, and, where relevant, physico-chemical properties (including isomerisation);
- identification data, including CAS number.

Vehicle (if appropriate):

- justification for choice of vehicle, if other than water.

Test animals:

- species/strain used;
- microbiological status of the animals, when known;
- number, age, and sex of animals (including, where appropriate, a rationale for the use of males instead of females);
- source, housing conditions, diet etc.

Test conditions:

- details of test substance formulation including details of the physical form of the

material administered;
- details of the administration of the test substance including dosing volumes and time of dosing;
- details of food and water quality (including diet type/source, water source);
- the rationale for the selection of the starting dose.

Results:

- tabulation of response data and dose level for each animal (i.e. animals showing signs of toxicity including mortality; nature, severity, and duration of effects);
- tabulation of body weight and body weight changes;
- individual weights of animals at the day of dosing, in weekly intervals thereafter, and at the time of death or sacrifice;
- date and time of death if prior to scheduled sacrifice;
- time course of onset of signs of toxicity, and whether these were reversible for each animal;
- necropsy findings and histopathological findings for each animal, if available.

Discussion and interpretation of results.

Conclusions.

LITERATURE

(1) Roll R., Höfer-Bosse Th. And Kayser D. (1986). New Perspectives in Acute Toxicity Testing of Chemicals. Toxicol. Lett., Suppl. 31, 86.

(2) Roll R., Riebschläger M., Mischke U. and Kayser D. (1989). Neue Wege zur Bestimmung der akuten Toxizität von Chemikalien. Bundesgesundheitsblatt 32, 336-341.

(3) Diener W., Sichha L., Mischke U., Kayser D. and Schlede E. (1994). The Biometric Evaluation of the Acute-Toxic-Class Method (Oral). Arch. Toxicol. 68, 559-610.

(4) Diener W., Mischke U., Kayser D. and Schlede E. (1995). The Biometric Evaluation of the OECD Modified Version of the Acute-Toxic-Class Method (Oral). Arch. Toxicol. 69, 729-734.

(5) Diener W., and Schlede E. (1999) Acute Toxicity Class Methods: Alternatives to LD/LC50 Tests. ALTEX 16, 129-134.

(6) Schlede E., Mischke U., Roll R. and Kayser D. (1992). A National Validation Study of the Acute-Toxic-Class Method – An Alternative to the LD50 Test. Arch. Toxicol. 66, 455-470.

(7) Schlede E., Mischke U., Diener W. and Kayser D. (1994). The International Validation Study of the Acute-Toxic-Class Method (Oral). Arch. Toxicol. 69, 659-670.

(8) OECD (2000) Guidance Document on Acute Oral Toxicity. Environmental Health and Safety Monograph Series on Testing and Assessment No 24.

(9) OECD (2000) Guidance Document on the Recognition, Assessment and Use of Clinical Signs as Humane Endpoints for Experimental Animals Used in Safety Evaluation Environmental Health and Safety Monograph Series on Testing and Assessment No 19.

(10) OECD (1998) Harmonized Integrated Hazard Classification System For Human Health And Environmental Effects Of Chemical Substances as endorsed by the 28th Joint Meeting of the

Chemicals Committee and the Working Party on Chemicals in November 1998, Part 2, p. 11 [http://webnet1.oecd.org/oecd/pages/home/displaygeneral/0,3380,EN-documents-521-14-no-24-no-0,FF.html].

(11) Lipnick R L, Cotruvo, J A, Hill R N, Bruce R D, Stitzel K A, Walker A P, Chu I; Goddard M, Segal L, Springer J A and Myers R C (1995) Comparison of the Up-and Down, Conventional $LD_{50,}$ and Fixed Dose Acute Toxicity Procedures. Fd. Chem. Toxicol 33, 223-231.

(12) Chan P.K. and A.W. Hayes. (1994). Chap. 16. Acute Toxicity and Eye Irritancy. *Principles and Methods of Toxicology*. Third Edition. A.W. Hayes, Editor. Raven Press, Ltd., New York, USA.

ANNEX 1

DEFINITIONS

Acute oral toxicity refers to those adverse effects occurring following oral administration of a single dose of a substance, or multiple doses given within 24 hours.

Delayed death means that an animal does not die or appear moribund within 48 hours but dies later during the 14-day observation period.

Dose is the amount of test substance administered. Dose is expressed as weight of test substance per unit weight of test animal (e.g. mg/kg).

GHS: Globally Harmonised Classification System for Chemical Substances and Mixtures. A joint activity of OECD (human health and the environment), UN Committee of Experts on Transport of Dangerous Goods (physical–chemical properties) and ILO (hazard communication) and co-ordinated by the Interorganisation Programme for the Sound Management of Chemicals (IOMC).

Impending death: when moribund state or death is expected prior to the next planned time of observation. Signs indicative of this state in rodents could include convulsions, lateral position, recumbence, and tremor (See the Humane Endpoint Guidance Document (9) for more details).

LD50 (median lethal oral dose) is a statistically derived single dose of a substance that can be expected to cause death in 50 per cent of animals when administered by the oral route. The LD50 value is expressed in terms of weight of test substance per unit weight of test animal (mg/kg).

Limit dose refers to a dose at an upper limitation on testing (2000 or 5000 mg/kg).

Moribund status: being in a state of dying or inability to survive, even if treated (See the Humane Endpoint Guidance Document (9) for more details).

Predictable death: presence of clinical signs indicative of death at a known time in the future before the planned end of the experimen ; for example: inability to reach water or food. (See the Humane Endpoint Guidance Document (9) for more details).

ANNEX 2

PROCEDURE TO BE FOLLOWED FOR EACH OF THE STARTING DOSES

GENERAL REMARKS

1. For each starting dose, the respective testing schemes as included in this Annex outline the procedure to be followed.

- Annex 2 a: Starting dose is 5 mg/kg bw
- Annex 2 b: Starting dose is 50 mg/kg bw
- Annex 2 c: Starting dose is: 300 mg/kg bw
- Annex 2 d: Starting dose is: 2000 mg/kg bw

Depending on the number of humanely killed or dead animals, the test procedure follows the indicated arrows.

ANNEX 2a: TEST PROCEDURE WITH A STARTING DOSE OF 5 MG/KG BODY WEIGHT

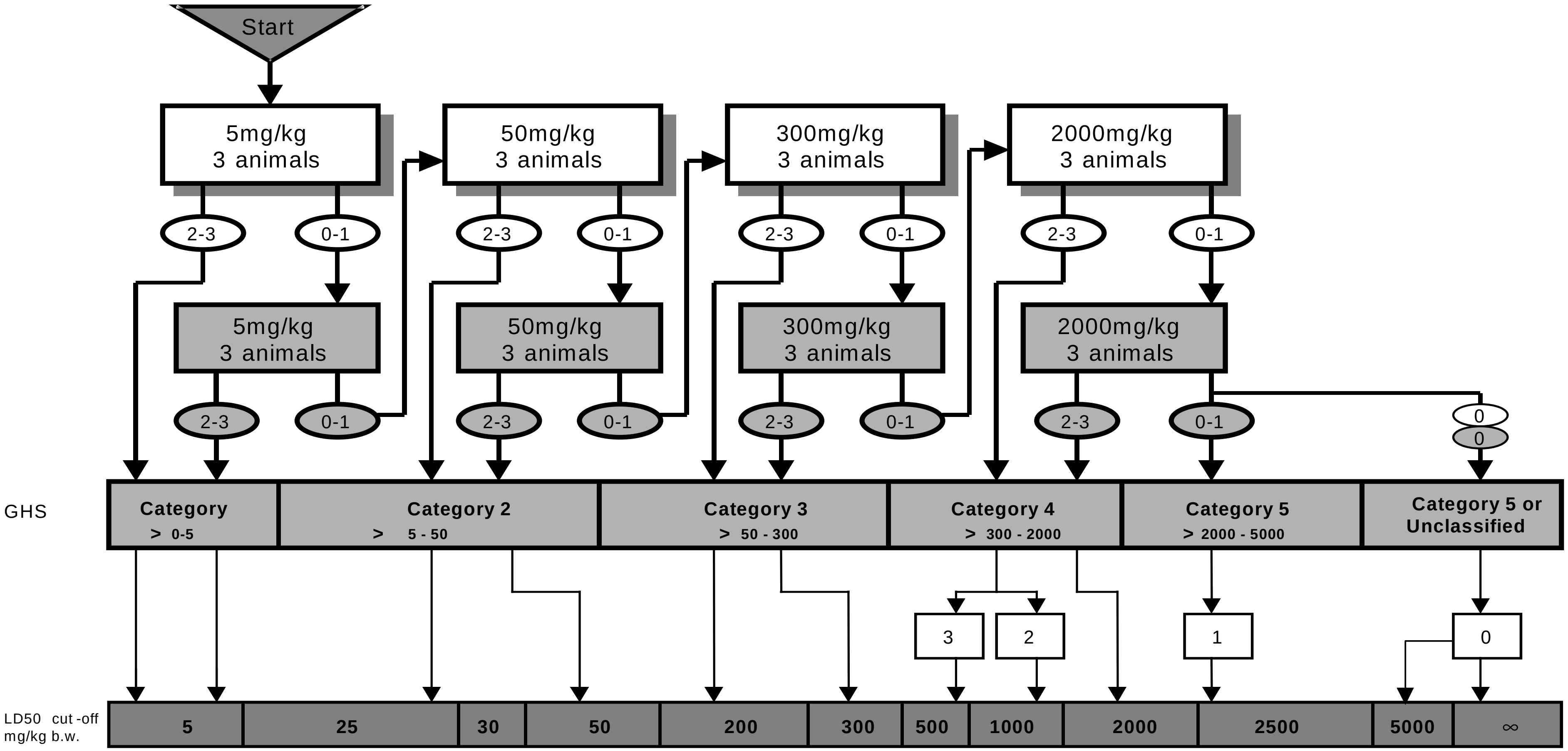

- per step three animals of a single sex (normally females) are used
- 0,1,2,3: Number of moribund or dead animals at each step
- GHS: Globally Harmonized Classification System (mg/kg b.w.)

- ∞ : unclassified
- Testing at 5000 mg/kg b.w.: see Annex 3

ANNEX 2b: TEST PROCEDURE WITH A STARTING DOSE OF 50 MG/KG BODY WEIGHT

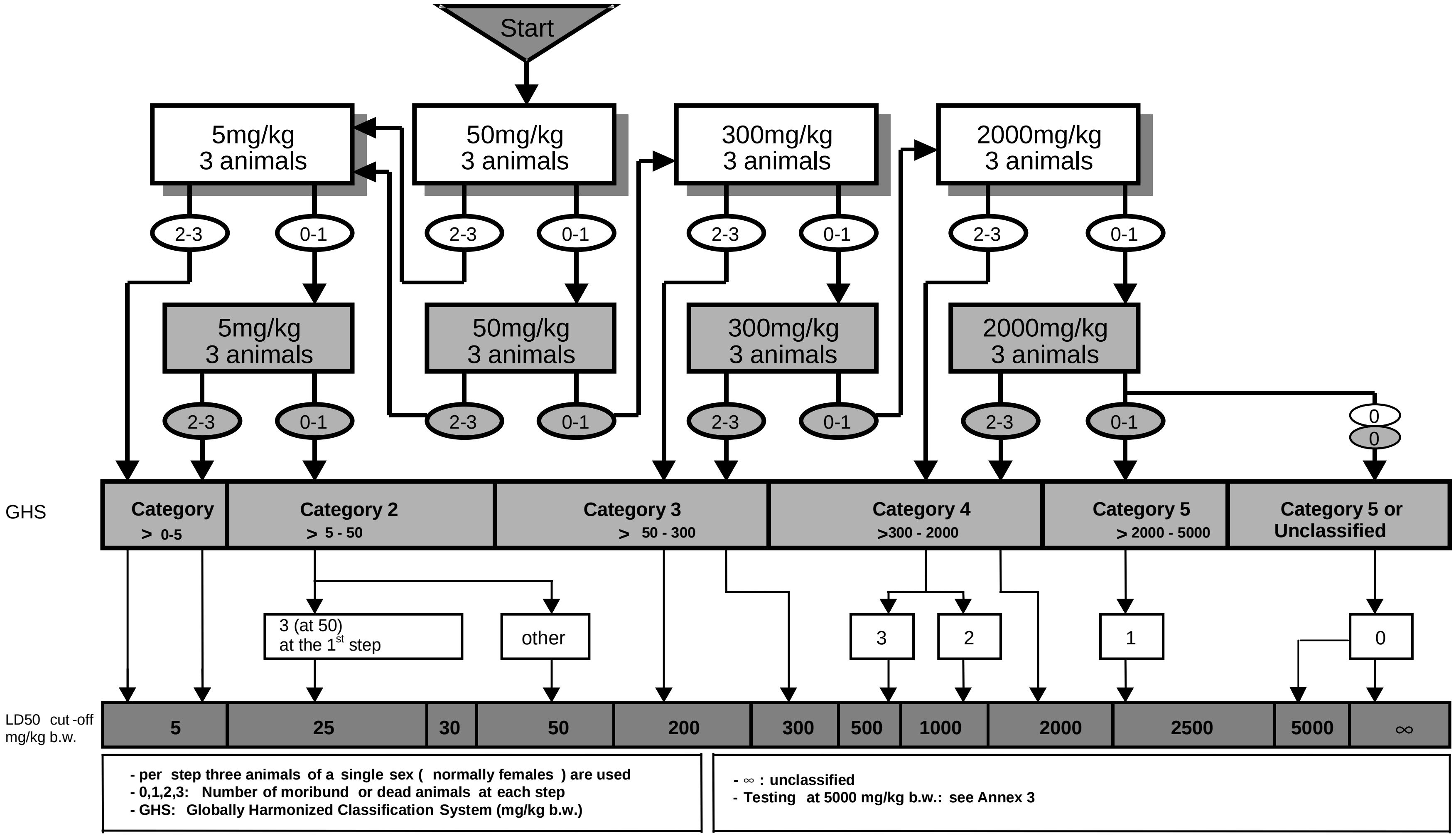

ANNEX 2c: TEST PROCEDURE WITH A STARTING DOSE OF 300 MG/KG BODY WEIGHT

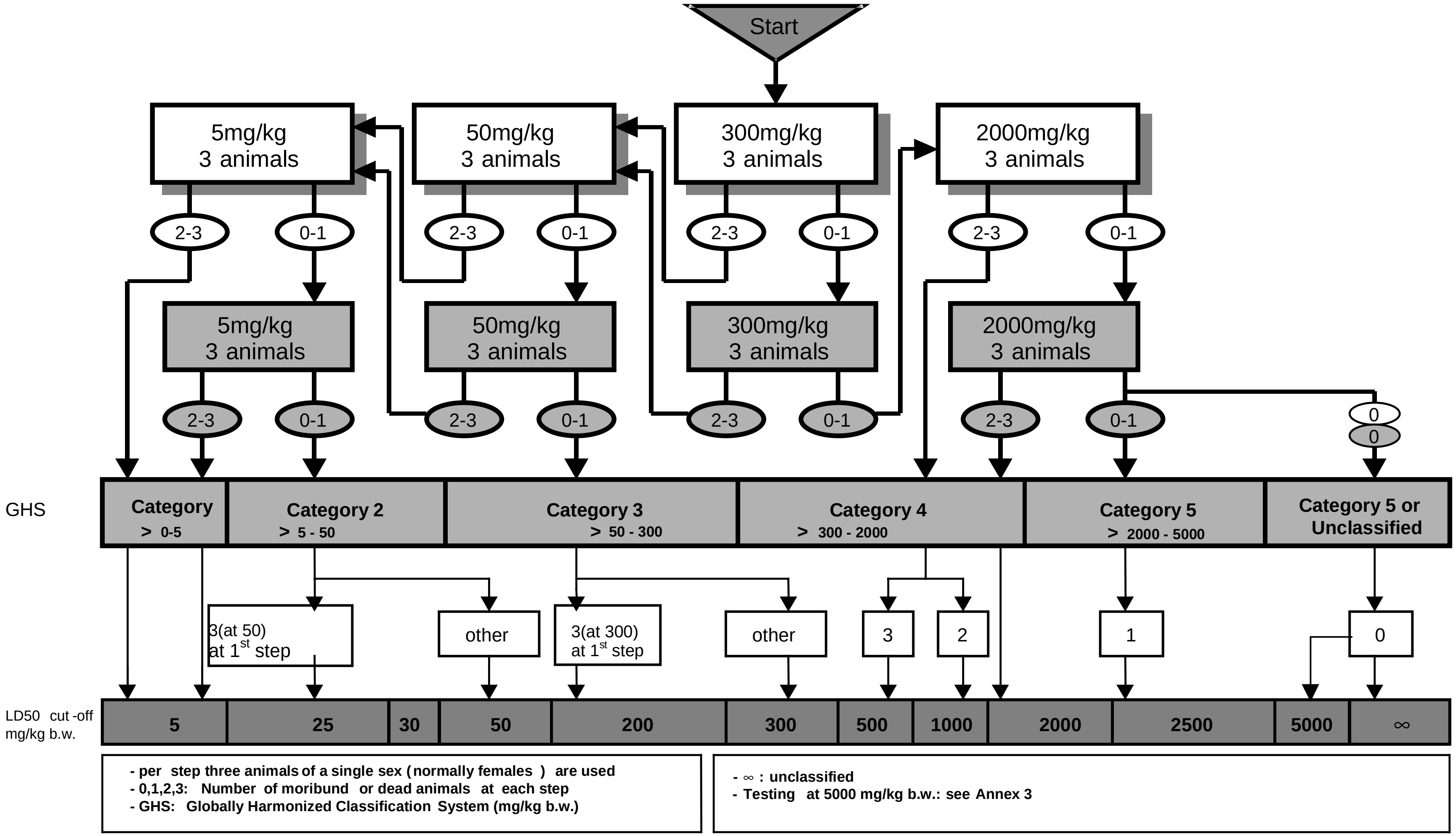

ANNEX 2d: TEST PROCEDURE WITH A STARTING DOSE OF 2000 MG/KG BODY WEIGHT

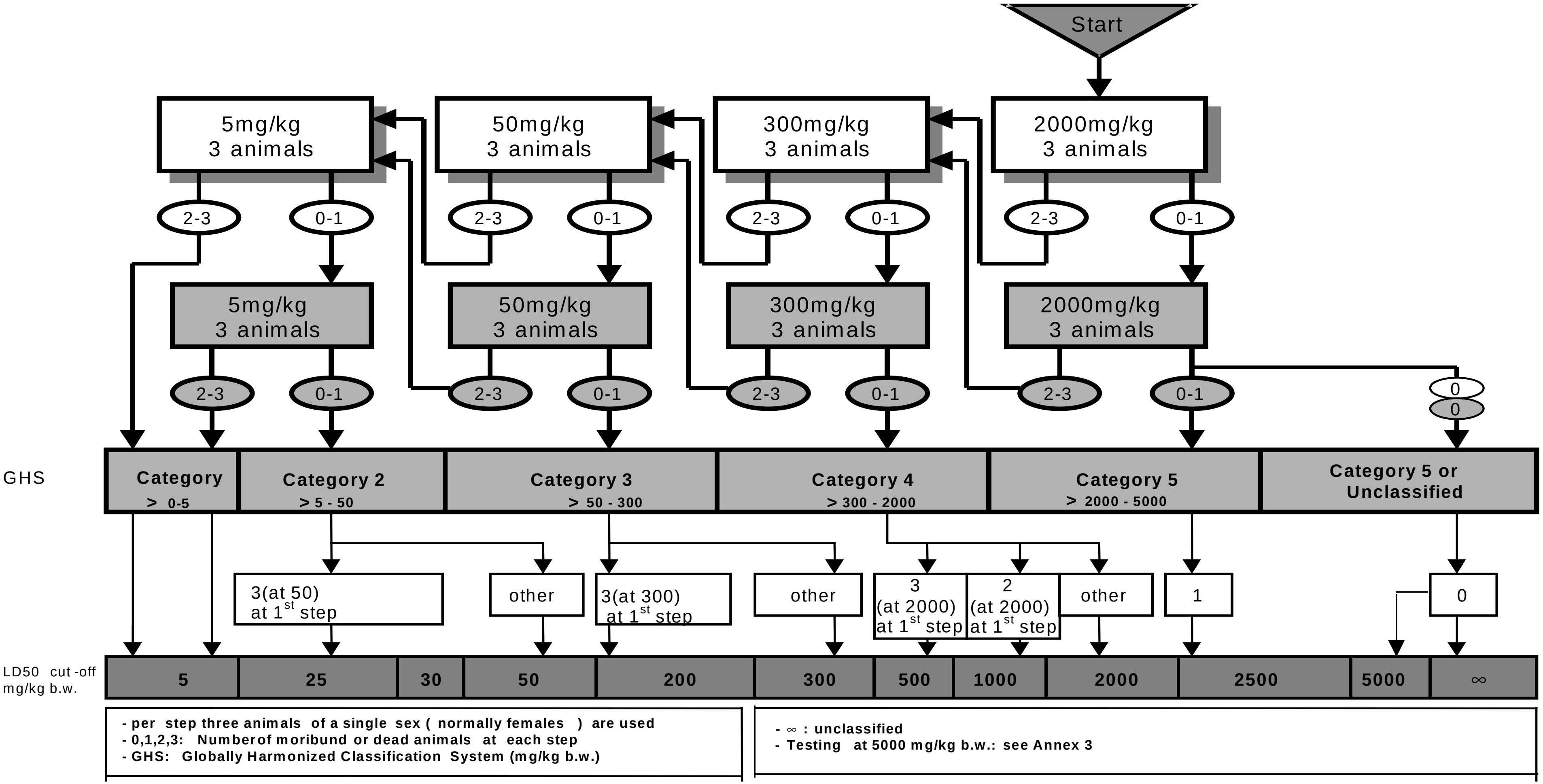

ANNEX 3

CRITERIA FOR CLASSIFICATION OF TEST SUBSTANCES WITH EXPECTED LD50 VALUES EXCEEDING 2000 MG/KG WITHOUT THE NEED FOR TESTING

1. Criteria for hazard Category 5 are intended to enable the identification of test substances which are of relatively low acute toxicity hazard but which, under certain circumstances may present a danger to vulnerable populations. These substances are anticipated to have an oral or dermal LD50 in the range of 2000-5000 mg/kg or equivalent doses for other routes. The test substance should be classified in the hazard category defined by: 2000mg/kg<LD50<5000mg/kg (Category 5 in the GHS) in the following cases:

a) If directed to this category by any of the testing schemes of Annex 2a-2d, based on mortality incidences;

b) if reliable evidence is already available that indicates the LD50 to be in the range of Category 5 values, or other animal studies or toxic effects in humans indicate a concern for human health of an acute nature.

c) Through extrapolation, estimation or measurement of data if assignment to a more hazardous category is not warranted, and

- reliable information is available indicating significant toxic effects in humans, or
- any mortality is observed when tested up to Category 4 values by the oral route, or
- where expert judgement confirms significant clinical signs of toxicity, when tested up to Category 4 values, except for diarrhoea, piloerection or an ungroomed appearance, or
- where expert judgement confirms reliable information indicating the potential for significant acute effects from the other animal studies.

TESTING AT DOSES ABOVE 2000 MG/KG

2. Recognising the need to protect animal welfare, testing of animals in Category 5 (5000 mg/kg) ranges is discouraged and should only be considered when there is a strong likelihood that results of such a test have a direct relevance for protecting human or animal health (10). No further testing should be conducted at higher dose levels.

3. When testing is required a dose of 5000mg/kg, only one step (i.e. three animals) is required. If the first animal dosed dies , then dosing procedes at 2000mg/kg in accordance with the flow charts in Annex 2. If the first animal survives, two further animals are dosed. If only one of the three animal dies , the LD50 value is expected to exceed 5000mg/kg. If both animals die, then dosing proceeds at 2000mg/kg.
